# Improving the Caesarean Decision by Robson Classification: A Population-Based Study by 5,323,500 Livebirth Data

**DOI:** 10.5334/aogh.2615

**Published:** 2020-08-17

**Authors:** H. Omer Tontus, Saniye Nebioglu

**Affiliations:** 1 Science and Letter Faculty, Molecular Biology and Genetics Department, Istanbul Technical University, TR; 2 Healthcare Services Department, Gynecology and Obstetrics Specialist, Public Hospital Directorate, TR

## Abstract

**Background:**

Caesarean section is a major obstetric intervention for saving lives of women and their newborns from pregnancy- and childbirth-related complications. C-Section rate is considered an important indicator for measuring obstetric services in any country, region, or institution. In many countries, based on population, all-cause C-Section rates have increased steadily during the past half century. The high and rising C-Section rate is certainly a cause for concern, and evidence-based information is needed as to how or why the C-Section rate has increased and what needs to be done. In this study, we tried to demonstrate how the Robson Classification can be used as a common starting point to audit caesarean deliveries.

**Objectives:**

Given the lack of a scientifically proven classification system to observe and compare caesarean rates, the WHO proposes adopting the Robson’s criteria-related grouping as an internationally applicable C-Section classification system.

**Methods:**

We conducted a retrospective study to look into the relation of Robson Criteria and C-Section. Our four years of study encompass 5,323,500 livebirths in Turkey and provide an important source of information for evaluating statistical data.

**Findings:**

We analysed pregnancies according to the percentage of live births in Robson’s groups and the caesarean rate within the Robson’s groups. In total, 2,764,373 pregnant women have undergone caesarean over 4 years with a 51.9% C-Section rate. According to our findings, as time progresses, the R5 group are expanding due to the caesarean sections groups R1–R4.

The R5 group C-Section rate increased regularly from 22.2% in 2013 to 24.3% in 2016. Caesarean sections performed in R1–R4 groups cause subsequent pregnancies of these women to result in caesarean section.

**Conclusions:**

Our results suggest the Robson classification system will help in analysing, screening, auditing, and comparing caesarean rates across different hospitals, countries, or regions and will help to create and implement effective strategies specifically to reach WHO recommended C-Section rates.

## Introduction

Caesarean section (C-Section) rate is an important indicator for measuring obstetric services in any country, region, or institution [1]. In many countries, C-Section rates have increased steadily during the past half century. Since 1985, the World Health Organization (WHO) considered the acceptable rate for C-Section to be between 10–15% [2]. The rates of C-Section have increased beyond 15% in many countries all over the world and have doubled in the last decade. When medically needed, a C-Section procedure can effectively prevent mortality or morbidity in both women and infants. While C-Section is required in some circumstances, the benefits of caesarean versus vaginal delivery for normal uncomplicated deliveries continue to be debated. C-Section delivery continues to result in increased maternal mortality, maternal and infant morbidity, and increased complications following deliveries, as well as increased need for finance, raising questions about the appropriateness of some C-Section that may not be medically required.

WHO emphasizes “Every effort should be made to provide C-Section to women in need, rather than striving to achieve a specific rate [2].” Infants can enter this world in one of two ways, either a vaginal birth or a C-Section, but the ultimate target is to safely give birth to a healthy baby. Above a recommended level, increasing the rate of C-Section is no longer associated with reduced mortality or morbidity in both baby and mother [2,3,4].

C-Section is a surgical procedure associated with short- and long-term risks that can affect the woman’s and infant’s health. In the last three decades, healthcare professionals, authorities, governments, and policymakers have conveyed their concern about the increasing C-Section rates and the potential negative consequences for maternal and newborn health.

WHO recommends that Robson’s classification be used as an appropriate C-Section classification system, because there is no scientifically proven classification system to observe and compare caesarean rates [2].

Ten-groups Robson classification provides an easy way of gathering information about the C-Section rate. Applying the classification helps to identify broad categories of pregnant women who can be targeted to reduce raising the C-Section rate [5].

Statistics from 150 countries show that C-Section worldwide reaches 18.6% of all births. Many European countries have managed to control or reduce C-Section rates over a period of time. Countries such as Norway, Finland, and Iceland are examples of countries where C-Section rates are around 15%. According to Pilar Betran and colleagues [6], while South America has the highest average caesarean rates in the world (42.9%), Africa has the lowest caesarean rates in (7.3%).

Turkey has the highest rate in the world, and the C-Section rate in many countries, including Turkey, continues to rise. According to the Organisation for Economic Co-operation and Development (OECD) numbers in 2016, Turkey is followed by Korea, Poland, Hungary, and Italy. The caesarean rate in Turkey is about 14% higher than Korea, the next highest country; the two countries have 53.2% and 39.4% C-Section rate, respectively.

The OECD explains the reasons for the increase in C-Section as follows: i) increase in nullipar older women, ii) multiple pregnancies as a result of assisted reproduction, iii) time management for both doctors and patients, iv) the preferences of some women [7].

Physicians are able to influence the choice of delivery mode, because they have better information than patients about birth physiology and possible complications. Lefevre’s “physician induced demand theory” predicts that physicians can shift the decision of pregnant women toward the one they prefer [8].

It will be possible to improve the “caesarean decision” by setting the areas of intervention and acting in the direction of guidance depending on Robson’s Criteria. The Robson’s criteria classify all deliveries into ten groups on the basis of five parameters: parity, the onset of labour, fetal presentation, number of fetuses, and gestational age [9,10].

## Materials and Methods

In this study, we aimed to use Robson’s classification to analyse the caesarean delivery rates of Turkey in four consecutive years. The caesarean rate is expressed as a percentage calculated by dividing the number of C-Section births by the total number of livebirths. We obtained the rates of C-Section from two important sources: i) routine vital statistics of TurkStat [11] and ii) yearly statistical reports of the Ministry of Health (MoH) [12,13]. The Turkish National Statistical Agency (TurkStat or TUIK) from which routine vital statistical data is taken is a reliable institution similar to Destatis (Germany) and the Office for National Statistics (the UK). TurkStat is the Turkish government agency commissioned with producing official statistics on Turkey’s population, resources, economy, society, and culture since it was founded in 1926. Official ruling document 2012–7413, published on May 14, 2012, made the evaluation of pregnant women according to Robson Classification and registration with the central database of the Women and Reproductive Health Department compulsory. In the first six months, on-site training and information sharing were made in hospitals. Since the beginning of 2013, Child Adolescent Women and Reproductive Health Unit (CEKUS) has controlled the keeping of records on the basis of provinces. Yearly statistical reports of the MoH clearly represent all hospital reports to the central data bank of Robson Classification data. Turkish identity cards for newborns can only be given with the birth certificate provided by the hospital where the birth is held. In this way, TurkStat can match the record between the birth certificate and the hospital data during the identity card application. In theory, it is foreseen that all hospital deliveries are recorded according to Robson Criteria.

WHO has proposed the Robson classification as an international standard. Thus, it may be possible to monitor the changes in caesarean delivery rates over time or to compare them on the basis of institutions or countries. Robson classification was used in over 33 million pregnancies in 31 countries following the WHO recommendation. In our study, every livebirth in four consecutive years was evaluated within the one group of the Robson Classification System. Caesarean rates for each Robson group and each hospital type, and annual variations of these rates, were calculated. Robson’s classification grouping is given in Table 1.

**Table 1:** The Robson’s Grouping system for caesarean deliveries.


Robson 1 (R1)	Nullipar, single cephalic, ≥37 weeks, spontaneous labour
Robson 2 (R2)	Nullipar, single cephalic, ≥37 weeks, induced
Robson 3 (R3)	Multipar (excluding previous caesareans), single cephalic, ≥37 weeks, spontaneous labour
Robson 4 (R4)	Multipar (excluding previous caesareans), single cephalic, ≥37 weeks, induced
Robson 5 (R5)	Previous caesarean, single cephalic ≥37 weeks
Robson 6 (R6)	All nullipar breeches
Robson 7 (R7)	All multipar breeches (including previous caesareans)
Robson 8 (R8)	All multiple pregnancies (including previous caesareans)
Robson 9 (R9)	All abnormal lies (including previous caesareans)
Robson 10 (R10)	All single cephalic, ≤36 weeks (including previous caesareans)


Induction of labour is the artificial initiation of labour before its spontaneous onset to deliver the feto-placental unit [14], and it is different from stimulating a labour that is demonstrating slow labour progress. According to WHO, any method of induction is valid including amniotomy, misoprostol, oxytocin, intracervical Foley catheter, or other. Women who enter labour spontaneously and then receive uterotonic agents or have an amniotomy to correct dystocias or to stimulate labour do not belong to the induced group and are classified as “spontaneous” onset of labour [15]. In our study, pregnant women were grouped within the Robson Classification according to WHO guidance.

In this article, Turkey’s current status in birth type decision was analysed and evaluated quantitatively on the basis of Robson’s classification. Frequency and percentages were used in the data analysis.

We also studied relationships between the Robson groups and hospital type (public or private). Study materials and data were obtained from the open-source data of the MoH.

## Results

Approximately 1.3 million births take place every year in Turkey. Our study group encompasses 5,323,500 livebirths over a four-year period. Turkey provides an important source of information for evaluating statistical data. Classification of all livebirths using Robson’s criteria is shown in Table 2. As we can see from the table, the biggest group for all years is R3, with 25.2%, followed by R5 and R1, with percentages of 23.4% and 22.9%, respectively.

**Table 2:** Number of livebirth and rates to Robson’s groups (n = 5,323,500).

	2013		2014		2015		2016		4 Years Total	

	(%)	Number	(%)	Number	(%)	Number	(%)	Number	%	Number

**Robson 1**	22.7	294,189	23	318,415	23.1	307,999	23.1	302,557	22.98%	1,223,160
**Robson 2**	10.8	139,967	10.6	146,748	10.3	137,333	9.7	127,048	10.35%	551,095
**Robson 3**	25.9	335,661	24.7	341,950	24.4	325,332	26.1	341,850	25.26%	1,344,793
**Robson 4**	7.6	98,495	7.8	107,984	7.2	96,000	7.1	92,994	7.43%	395,473
**Robson 5**	22.2	287,709	23.2	321,184	24.1	321,332	24.3	318,274	23.45%	1,248,500
**Robson 6**	2.6	3,696	2.6	35,995	2.6	34,667	2.3	30,125	2.53%	134,482
**Robson 7**	1.8	23,328	1.9	26,304	1.9	25,333	1.7	22,266	1.83%	97,231
**Robson 8**	1.4	18,144	1.4	19,382	1.3	17,333	1.3	17,027	1.35%	71,886
**Robson 9**	1.7	22,032	1.7	23,535	1.6	21,333	1.3	17,027	1.58%	83,927
**Robson 10**	3.3	42,768	3.1	42,917	3.5	46,667	3.1	40,603	3.25%	172,954
**Total**	**100**		**100**		**100**		**100**		**100**	
**Total R1-R4 groups**	67.0	868,311	66.1	915,097	65.0	866,664	66.0	864,449	66.02%	3,514,521
**Total C-section**	**51.4%**	**665,547**	**52.6%**	**727,609**	**53.6%**	**714,636**	**50.1%**	**656,582**	**48.1%**	**2,764,373**
**Total Vaginal Deliveries**	**48.6%**	**630,441**	**47.4%**	**656,804**	**46.4%**	**618,693**	**49.9%**	**653,189**	**48.1%**	**2,559,127**
**Total Live-Birth number**	**1,295,987**		**1,384,413**		**1,333,329**		**1,309,771**		**5,323,500**	

### 1 Results of distribution of livebirth and cesarean rates according to Robson Classification

When we look at Table 2, the figures for Robson’s groups show similar trends from 2013 to 2016. Overall, the percentage of R1–R4 groups changed from 67% in 2013 to 66% in 2016. Year by year, the number of pregnant women in the R5 group expanded due to caesarean procedures in the R1–R4 groups. The R5 rate increased regularly from 22.2% in 2013 to 24.3% in 2016.

Distribution of pregnancies delivered by caesarean section across Robson groups (number of C-Section/Number of livebirth %) is given in Table 3. As shown in Table 3, 2,764,373 pregnant women gave birth by caesarean over 4 years (51.9% C-Section rate). The highest C-Section rate was in the R5 group, with 1,209,958 of 1,248,500 women delivering by C-Section (96.9%). When delivery was medically induced (R2), the caesarean delivery rate almost doubled when compared to uninduced delivery (R1) as shown in Table 3. In total over four years, R2 and R4 (both are induced groups) had C-Section rates of 63.9% and 41.2%, respectively. Groups R1 and R3 have the same clinical condition with R2 and R4 except for induction. These two groups had 33.2% and 12.5% C-Section rates, respectively, over four years (Table 3). That means induction plays a critical role in the C-Section rate.

**Table 3:** C-Section rate in each group (number of C-Section/Number of Livebirth %) (livebirth n = 5,323,500).

	2013		2014		2015		2016		4 Years Total	

	Number of Caesarean	Caesarean rate %	Number of Caesarean	Caesarean rate %	Number of Caesarean	Caesarean rate %	Number of Caesarean	Caesarean rate %	Number of Caesarean	Caesarean rate %

**Robson 1**	100,024	34	108,261	34	106,568	34.6	91,372	30.2	406,225	33.2%
**Robson 2**	88,739	63.4	93,625	63.8	91,189	66.4	78,643	61.9	352,196	63.9%
**Robson 3**	43,972	13.1	46,505	13.6	41,317	12.7	36,920	10.8	168,714	12.5%
**Robson 4**	40,580	41.2	44,489	41.2	41,856	43.6	36,175	38.9	163,100	41.2%
**Robson 5**	277,352	96.4	311,869	97.1	311,692	97	309,044	97.1	1,209,958	96.9%
**Robson 6**	30,730	91.2	32,323	89.8	31,269	90.2	25,998	86.3	120,321	89.5%
**Robson 7**	20,342	87.2	22,069	83.9	22,116	87.3	19,683	88.4	84,210	86.6%
**Robson 8**	15,567	85.8	17,715	91.4	16,311	94.1	15,767	92.6	65,360	90.9%
**Robson 9**	19,586	88.9	20,452	86.9	18,859	88.4	15,086	88.6	73,983	88.2%
**Robson 10**	28,654	67	30,299	70.6	33,460	71.7	27,894	68.7	120,308	69.6%
**TOTAL Caesarean**	**665,546**	**51.4%**	**727,609**	**52.6%**	**714,636**	**53.6%**	**656,582**	**50.1%**	**2,764,373**	**51.9%**
**Vaginal Deliveries**	**630,441**	**48.6%**	**656,804**	**47.4%**	**618,693**	**46.4%**	**653,189**	**49.1%**	**2,559,127**	**48.1%**
**TOTAL Livebirth**	**1,295,997**	**100%**	**1,384,413**	**100%**	**1,333,329**	**100%**	**1,309,771**	**100%**	**5,323,500**	**100%**

The percentage of all caesarean deliveries by Robson group is given in Table 4. As shown in Table 4, the largest C-Section group is R5 with 43.8% of all C-Section cases. The C-Section ratio in the R5 groups increased from 41.7% in 2013 to 47.1% in 2016. In the mean of 4 years, the rate of C-Section in patients in the R5 group was 43.8%.

**Table 4:** Relative contribution made by each Robson group to overall C-Section rate (number of C-Section in each group/number of total yearly C-Section %) (4 years total livebirth n = 2,764,373).

	2013	2014	2015	2016	4 Years Total

**Robson 1**	15.0%	14.9%	14.9%	13.9%	14.7%
**Robson 2**	13.3%	12.9%	12.8%	1.,0%	12.7%
**Robson 3**	6.6%	6.4%	5.8%	5.6%	6.1%
**Robson 4**	6.1%	6.1%	5.9%	5.5%	5.9%
**Robson 5**	41.7%	42.9%	43.6%	47.1%	43.8%
**Robson 6**	4.6%	4.4%	4.4%	4.0%	4.4%
**Robson 7**	3.1%	3.0%	3.1%	3.0%	3.0%
**Robson 8**	2.3%	2.4%	2.3%	2.4%	2.4%
**Robson 9**	2.9%	2.8%	2.6%	2.3%	2.7%
**Robson 10**	4.3%	4.2%	4.7%	4.2%	4.4%
**TOTAL**	100%	100%	100%	100%	100%

### 2 Robson Classification cesarean rates in hospital groups

Table 5 shows the caesarean percentages of Robson’s groups from 2013 to 2016. Among the pregnant women who apply to hospitals, private hospitals have the highest C-Section rates. In 2016, the caesarean section rate in R1 was 30.2%; whereas, it reached 61.9% in R2 due to induction. This suggests that how and when to intervene in labour should be re-examined or delivery guidelines should be revised.

**Table 5:** Caesarean rates of Robson’s groups by hospital types (%).

	Hospital Groups	Robson 1	Robson 2	Robson 3	Robson 4	Robson 5	Robson 6	Robson 7	Robson 8	Robson 9	Robson 10

**2013**	**Public State**	21.2	44.1	7.4	26.6	95.9	93.1	83.6	80.0	91.5	50.4
	**Private**	48.1	76.5	27.2	59.1	96.7	90.5	88.6	89.7	88.3	80.3
	**Universit**y	40.5	68.1	20.9	52.3	97	90.3	89.1	84.0	88.5	64.1
	**Caesarean rate**	**34.0**	**63.4**	**13.1**	**41.2**	**96.4**	**91.2**	**87.2**	**85.8**	**88.9**	**67.0**
**2014**	**Public State**	19.8	46.4	7.6	26.5	97.3	87.1	74.3	86.3	80.9	56.3
	**Private**	51.9	75.8	29.3	58.4	96.9	90.8	88.5	94.4	88.7	80.4
	**Universit**y	39.4	64.6	22.2	60.5	97.8	90.2	88.1	91.6	90.2	69.1
	**Caesarean rate**	**34.0**	**63.8**	**13.6**	**41.2**	**97.1**	**89.8**	**83.9**	**91.4**	**86.9**	**70.6**
**2015**	**Public State**	19.0	51.6	6.5	30.6	97.5	86.3	83.5	87.6	80.3	59.3
	**Private**	53.6	75.7	29.9	56.9	96.8	92.3	89.0	98.5	90.1	80.8
	**Universit**y	47.1	70.3	37.4	58.3	95.3	83.2	87.4	95.0	88.2	72.7
	**Caesarean rate**	**34.6**	**66.4**	**12.7**	**43.6**	**97.0**	**90.2**	**87.3**	**94.1**	**88.4**	**71.7**
**2016**	**Public State**	18.3	50.4	6.9	29	97.5	79.9	87.2	87.6	81	57.5
	**Private**	52.6	74.4	27.9	56.6	96.6	91.2	89.5	97.6	91.1	82
	**Universit**y	47	71.6	32.3	60	95.38	83.6	87.4	95.3	86.4	71.2
	**Caesarean rate**	**30.2**	**61.9**	**10.8**	**38.9**	**97.1**	**86.3**	**88.4**	**92.6**	**88.6**	**68.7**

Groups R1 to R4 in all years in the private healthcare sector had higher C-Section rates. A significant proportion of the pregnancies were in Robson groups 1–4, which are vaginal birth candidates except for some clinical necessities. As seen in Table 5, for 2016, the pregnant women in R1 and R2 gave birth mostly by caesarean in private hospitals at the rate of 52.6% and 74.4%, respectively. C-Section birth rates in public hospitals of the same groups were 18.3% and 50.4%, respectively.

As can be seen from Table 5, in all years the group with the lowest caesarean rate is R3 and the group with the highest rate is R5. As four years of consecutive data indicates, each primary C-Section birth carries the potential caesarean birth risk on the next pregnancy. Because of that, every C-Section decision must be recorded with certain criteria depending on guidelines.

In addition, when each group is compared within hospital type, the differences are striking and clear. In R1, where labour started spontaneously, the proportion of C-Section in public hospitals was 18.3% in 2016; whereas, it reached to 52.6% in the private sector. As seen in Table 5, the C-Section rate in the private sector, which are for-profit facilities, was 50%.

This finding indicates that preferences of institutions, patients, physicians, or other healthcare professionals are important in making medical decisions for the delivery method.

Among women who have previously delivered by vaginal birth, it is difficult to make a scientific explanation for the difference in the caesarean rate between the public and private sectors, especially for the R3 group (6.9% and 27.9%, respectively, in 2016). The rate of caesarean section in R5 (former caesarean section) group was just over 97% in 2016.

## Discussion

The high and rising C-Section rate is certainly a cause for concern, and evidence-based information is needed about how or why the C-Section rate has increased and what needs to be done. In this study, we tried to demonstrate how the Robson classification can be used as a common starting point to audit caesarean deliveries. We also compared C-Section rates in health-care facilities using the Robson classification system and found that C-Section rates in the R5 group increased over time. And we noticed that every caesarean in groups R1–R4 added new C-Section candidates in their subsequent pregnancies to group R5, which already had the highest C-Section rate (96.9% four-year average).

On the one hand, and perhaps most importantly, the mother’s decision is affected by incorrect and/or incomplete information and other environmental factors. To impact positively on this factor in Turkey as a reliable data source, a unique “video on demand” website (www.annevebebek.gov.tr) began broadcasting. Video resources based on expert opinions can be an important element of intervention in supporting mothers’ knowledge competence in pregnancy and childbirth.

On the other hand are health professionals, who can seriously affect the mother’s choice of mode of birth because they have much more information than pregnant women. The physician-based demand theory predicts physicians can bring their decision over the mother’s preferred decision [8].

Regardless of the circumstances and reasons, the MoH established and coordinates rules for lowering cesarean rates. Information has been organized to cover all related parties. Training and informative guidance activities are carried out for pregnant women and their relatives, doctors, and other health professionals. In addition, sanctions or incentives for institutions may be implemented by the MoH according to caesarean rates and indications for caesarean based on the guidelines.

Common medical indications for a caesarean section include abnormal presentation, fetal distress, umbilical cord prolapses, placenta previa, uterine rupture, failed labour induction, macrosomia, preeclampsia, and previous caesarean section. Without guidelines with specific standards, some of these indications are subjective and variable for decision-makers (physician or other healthcare professionals, including midwives) [16,17]. For a more detailed assessment with an evidence-based approach, the effects of each step of service given to pregnant women based on the caesarean indications until the date of birth should be examined by a doctor on an institution basis [18].

Our analysis showed that in all three hospital types, women who previously had caesarean section (R5) significantly increased the total cesarean rate. As a matter of fact, the caesarean rate in the R5 group remained above 95% over four consecutive years. WHO analysis clearly shows if caesarean section rates increase, more women are in need of repeat C-Section, as indicated by the increasing contribution of the R5 group to the overall caesarean rates over time [10].

Some researchers have indicated that the nulliparous population is the largest contributor to the overall cesarean rate [10]. We found similar findings, as seen in Table 3. This situation is especially noticed in the private sector.

According to Lefevre, four factors play a role in a physician’s preference for caesarean delivery. These are financial incentives (C-Section is more profitable), time management (vaginal birth requires more time), fear of malpractice, and the desire to devote time to their own social lives [8].

Again, the attitudes of health facilities, especially private hospitals, can be regarded as favouring caesarean section, and health workers play a decisive role in the choice of birth method. Furthermore, the socio-cultural infrastructure of both physicians and patients can act as a decisive factor in the decision-making process. Supporting the view that the private sector could increase C-Section rates for higher profits based on surgical procedures, the highest C-Section rate was found in for-profit private-sector facilities [19,20].

A worldwide study that included 24 countries and 373 health facilities found that, compared with vaginal delivery, elective C-Section without medical indication was concomitant with an augmented risk of maternal complications, even maternal death, admission to intensive care unit, transfusion, and hysterectomy [21].

Regulations encouraging vaginal birth require a change in pregnant women’s social attitudes and/or healthcare professionals’ occupational habits. In addition, even for patients planning a normal vaginal delivery, the delivery method may change in favour of caesarean section based on the preferences of hospitals and/or physicians. Therefore, it may require a long time to reach targeted results.

With appropriate caesarean indications, the vaginal delivery rights of patients and the foetuses should be protected. The Robson 1 and 2 groups, which are low-risk individuals, should be prioritized with realistic targets in order to lower the caesarean rates [22]. In order to increase this awareness, it is necessary to work together with health facilities, non-governmental organizations, medical chambers (such as the Society of Gynaecology and Obstetrics or National Medical Association) to “improve the quality of the caesarean decision.” In addition, caesarean rates can be reduced by generating solutions, such as the Transform Maternity Care Program in Los Angeles, the Quarisma Project in Canada and Spain, and the Linköping University Project in Sweden, taking into account the workflow procedures of all institutions [23,24,25,26,27,28].

Studies aimed at reducing caesarean rates absolutely must be based on scientific bases, statistical data, and information produced in this way should be expressed in national congresses and symposiums to raise awareness. Probably the most important thing for the process is to study how to reduce caesarean rates through pilot programs and to demonstrate this by creating a successful regional or institutional model [23,25]. In a high-quality review of global ceasarean delivery rates and newborn outcomes, Molina and colleagues reported that a caesarean delivery rate of 15% to 20% was associated with optimal newborn outcomes and a relatively low maternal caesarean delivery rate [29]. While they reported that when caesarean delivery rates were <15% there appears to be an increase in adverse newborn outcomes, including newborn mortality, WHO has not revised their target of 10% to 15% since 2015. WHO also stated that if the caesarean rate goes above 10%, there is no evidence that mortality rates improve [2].

In the low-risk group, birth should be expected to start spontaneously. As suggested by the American College of Obstetricians and Gynecologists (ACOG), two factors have been important to reduce C-Section rates: (1) “Don’t induce birth before 39 weeks” and (2) “wait for the active birth phase to reduce un-progressing labor diagnosis [9,30,31].” On the other hand, Grobman and colleagues reported lower increases of caesarean rates after induction when compared to our study [30]. Labour induction time in their multicenter trial started at 39 weeks 0 days to 39 weeks 4 days, and they didn’t group the women using the Robson classification. They also didn’t include post-term pregnancies in their study. Their study group is not compatible with Robson 1 to Robson 4. Because gestational weeks in later groups are 37 weeks or greater, our study group’s results are different than Grobman and colleagues’ results.

The United States plans to reduce C-Section rate in the low-risk group to an average of 23.9% in the WHO-designated Healthy People 2020 target [16]. This rate is above the 15% prescribed by WHO for any part of the world [2]. The fact that countries form targets based on their own reality will increase the success rate of their work in favour of vaginal delivery. In the United States, from the mid-1980s to the mid-1990s, an increase in vaginal birth after caesarean (VBAC) delivery was seen along with a concomitant decrease in caesarean delivery rate. But over time, the number of reported significant complications and accompanying malpractice suits caused a decrease in VBAC [32]. As seen in Table 3, healthcare professionals in Turkey still stand behind the dictum, “once a caesarean, always a caesarean,” and the number of patients undergoing VBAC delivery remain very low.

Maternity services in England have set out a woman’s right to choose a caesarean section even if there is no clinical need, and clinicians offer to counsel on the decision to help them understand the relative risks. National Institute for Clinical Excellence (NICE) guidelines say that the formalization of the right to choose and be counselled will, in fact, reduce caesarean rates as women will get better advice. Only 25% of livebirths are now done by caesarean in England [33].

Some studies indicate that average total charges per childbirth depend on many factors, such as insurance policies and delivery method [34,35]. In Turkey, if a hospital prefers C-Section, public reimbursement pays much more than vaginal delivery. Thus, doctors and hospitals earn more. Therefore, the payment system reform for delivery is crucial to reduce the C-Section rate. The medical insurance institutions and other payers must introduce a payment standard based on the objective indications to strengthen physicians’ comprehensive skills on delivery indications.

In our study, we found that Robson classification provides important standard data for the evaluation of caesarean rates and caesarean decisions of hospitals, physicians, and even regions or countries, similar to Vogel’s findings [10]. The expanding R5 group also signals that women who have previously had a caesarean section are an increasingly important determinant of overall C-Section rates.

In Table 6, we summarize a list of interventions for Turkey about directing all parties in favour of vaginal birth. As can be seen from the table, MoH should play a role in all of the basic principles, such as training; setting standards, incentives, and interventions; reorganizing the staff structure; coordinating infrastructure of the birth process management. Other important institutions and organizations with important roles to reduce caesarean rates are specialty associations, universities, and institutional bodies.

**Table 6:** Top 10 recommended interventions for reducing the caesarean rate in Turkey.

	What to do	Why to do	Who to do

**1**	Measures and incentives should be developed for the private sector.	To reduce private sector caesarean rate to the public hospital level.	MoH Ministry of Finance Reimbursement Agencies
**2**	“The Vaginal Delivery Right” should be discussed and an agenda should be created.	To raise awareness for all parties (healthworker-pregnants-families).	MoH Universities NGOs
**3**	Healthcare providers (hospitals/obstetricians) should be motivated to create and lead corrective actions. Feedback to both the physician and the institution should be made about their caesarean rate by MoH.	To support and divert healthcare professionals’ and institutions’ motivation towards vaginal delivery with feedback	MoH Specialty Boards Specialty associations
**4**	It should be ensured that the residents who are on obstetrics training in a hospital with less than 500 vaginal deliveries per year spend one year of their education in hospitals with more vaginal deliveries.	To train future obstetricians with more experience in vaginal delivery.	MoH Universities Specialty Boards Specialty Associations
**5**	Regional obstetrical reference centers should be determined for vaginal birth after caesarean (VBAC).	To reduce the secondary caesarean rate due to previous caesarean indication	MoH
**6**	Midwifery should be encouraged and pregnancy coach (doula) should be included in the system.	To increase the number of healthcare professionals in favour of vaginal birth	MoH Universities Policy makers
**7**	Antenatal educational activities for expectant about pregnancy, birth, and postpartum periods should be strengthened.	Guidance of expectant to vaginal delivery by educating that pregnancy process is a natural cycle, vaginal birth is more natural, and it is possible to switch to natural life cycle easily afterwards.	Public Hospital Private Hospital Practitioners MoH NGOs
**8**	The use of “Mother and Baby web-TV (www.annevebebek.gov.tr)” which is still live should be supported and its content should be enriched.	To provide access to reliable information to expectant mothers regardless of time and location	MoH Universities NGOs Private Hospital
**9**	Guidelines for the birth process should be updated and compliance should be followed on the basis of institution or department.	To evaluate the reason for the caesarean decision and also to obtain statistical data for follow-up	Universities MoH
**10**	Supporting and providing legal counselling to healthcare professionals in malpractice cases encountered during and after birth	Since the birth process is considered risky by healthcare workers and they are afraid of malpractice cases that may arise due to problems that may arise due to this, institutions take a stance in favour of caesarean.	Policy Makers MoH Private Sector Specialty Associations

## Conclusion

The frighteningly high C-Section rate calls for monitoring indications of all C-Sections in public and private facilities [3]. The C-Section rate in Turkey is much higher than any country as well as the WHO global recommendations. Routine monitoring of clinical indications of C-Section in institutions with certain criteria, such as Robson’s classification, is needed to ensure optimal use of the procedure.

Caesarean section, one of the most frequently performed surgical procedures, is on the rise globally. Our findings indicate that preferences of institutions, patients, physicians, or other healthcare professionals are important in making medical decisions for the delivery method. On the way to improving the caesarean decision, the central authorities or MoH may be the driving force for every institution. However, it is important to show how it can be done and that it can be done with pilot studies to ensure that academics see the issue as a problem that needs to be solved.

We noticed that standards for lowering the caesarean rate have not been shared properly with all parties, such as for physicians, hospitals, and patients, in Turkey. Because the standards are not properly published and shared, hospitals and doctors cannot assess their own practice. While most of the physicians accept that caesarean rates are high, many of them think their practice is adequate, scientific, and accurate.

To ensure one of the five indications (fetal distress, non-progressive or obstructed labour, head-pelvis incompatibility, preeclampsia, macrosomia) is seen, clinical reasoning based on scientific evidence is required for each caesarean section to be performed in the R1, R2, R3, and R4 groups.

Change is difficult if health care providers serve based on their own preferences. Therefore, it is necessary to establish evidence-based standards to improve the caesarean decision. According to Imai and Shingo, a process must first be stabilized then standardized before being improved [36]. Because of the lack of standardized processes in child-birth care, the most important factor for lowering the C-Section rate is to get the physicians, nurses, midwives, hospitals, and even policymakers to agree on what is the best way to deliver birth-care. As the guiding effect of doctors and other health professionals is known, it is necessary to ensure that experts contribute to the development of standards to implement standards in obstetric care processes.

Scandinavian countries effectively keep their focus on higher rates of vaginal births by having strict guidelines. In countries with access to high-quality maternity care, it is possible to reduce C-Section numbers.

The problem of higher rates of C-Section arising in hospitals and due to healthcare professionals should be identified and a step-by-step solution for each obstacle should be identified. The most important factor in reaching the targets is that the service providers (both the institutions and the healthcare professionals) must be part of the solution.

To decrease the C-Section rate by normalising vaginal birth, policymakers and health authorities need to develop a culture with a systematic approach that supports and promotes vaginal deliveries. A well-designed campaign with the support of all parties can reduce unnecessary caesarean birth, especially in nullipar women.

Our results suggest that Robson’s grouping is the best option to fulfil current international and local or regional needs and that efforts to develop an internationally applicable C-Section classification would be appropriate to build upon this method. The use of Robson classification as a global caesarean classification system will help in analysing, screening, auditing, and comparing caesarean rates across different hospitals, countries, or regions and will help to create and implement effective strategies specifically to reach WHO recommended C-Section rates [37]. Multipar women who have previously had a C-Section are an increasingly important element of overall C-Section numbers. Strategies or campaigns to decrease the rate of the C-Section and to improve the caesarean decision should include avoidance of medically unnecessary primary C-Section and improved case selection for induction. As repeat C-Section is a dominant cause, reduction of primary C-Section should be given priority.

As the last word, we conclude that evidence-based interventions and programmes or health-promotion campaigns to reduce both primary and repeat caesarean sections are needed.

## Data Accessibility Statement

I can confirm data and materials available via email contact, if needed. Materials described in the article are freely available to any scientist wishing to use them for non-commercial purposes, without breaching confidentiality.
